# Incidence Rate of Stroke in Peru

**DOI:** 10.17843/rpmesp.2021.383.7804

**Published:** 2021-12-20

**Authors:** Antonio Bernabé-OrtizG, Rodrigo M. Carrillo-Larco

**Affiliations:** 1 CRONICAS Centro de Excelencia en Enfermedades Crónicas, Universidad Peruana Cayetano Heredia, Lima, Peru; 2 Universidad Científica del Sur, Lima, Peru; 3 Department of Epidemiology and Biostatistics, School of Public Health, Imperial College London, London, United Kingdom

**Keywords:** Stroke, Ischemic Stroke, Hemorrhagic Stroke, Subarachnoid Hemorrhage, Epidemiology, Incidence, Peru (source: MeSH NLM)

## Abstract

**Objective:**

To determine the incidence of stroke, overall and by sub-types, in Peru between 2017 and 2018.

**Materials and Methods:**

Analysis of hospital morbidity data obtained from SUSALUD (open data). Using the ICD-10 codes, the following were studied: subarachnoid hemorrhage (I60), atraumatic intracerebral hemorrhage (I61), cerebral infarction (I63), and unspecified stroke (I64). The crude and age-standardized incidence was calculated according to the population of the World Health Organization and using the national projected population number of people according to year, age and sex as the denominator.

**Results:**

In 2017, a total of 10,570 stroke cases were recorded, whereas, in 2018, there were 12,835 cases. Ischemic events were more frequent in both years. Regardless of stroke subtype and year, men were more affected than women. In the 35+ year-old population, an increase in the crude incidence of total stroke was observed between 2017 and 2018, from 80.9 to 96.7 per 100,000 person-years. The age-standardized incidence showed the same trend, but in a greater magnitude: from 93.9 to 109.8 per 100,000 person-years. Ischemic stroke was the one that increased the most, with an age-standardized rate in people aged 35+ years of 35.2 in 2017 and 46.3 per 100,000 person-years in 2018.

**Conclusions:**

The incidence of stroke is high in Peru. Ischemic cases are the most frequent and they disproportionately affect men. Our results suggest the need for a surveillance system to robustly quantify the incidence of these cases and understand their determinants.

## Introduction

Worldwide, cardiovascular events are the leading cause of death ^(1)^. Among them, strokes are one of the leading reasons for disability and mortality ^(2)^. By 2016, about 5.5 million deaths were estimated to be secondary to stroke ^(3)^, and the incidence rate of stroke in low- and middle-income countries exceeds the incidence in high-income countries ^(4)^.

Although the incidence of stroke has been decreasing since 1990, more than 80% of stroke cases occur in low-income countries ^(5)^, with an estimated incidence of 117 per 100,000 person-years, ranging from 73 to 165 per 100,000 person-years ^(4)^. However, surveillance of this condition is limited in these countries, as is the case of Peru. For example, the incidence of stroke in the population, overall and by stroke subtype, is unknown, since most of the information comes from mathematical models based on international data which are then applied to our context.

According to the literature, there are several ways of determining the incidence of stroke in the population. Thus, some authors have determined the incidence of stroke using longitudinal cohort studies ^(6)^, others have done so by establishing stroke registry systems in certain regions of certain countries ^(7)^, or by using self-report of neurological symptoms compatible with stroke (including the use of imaging or specialized evaluation) occurring in the year prior to the evaluation ^(8)^. However, few studies have used national stroke registries to determine not only the incidence of stroke, but also the incidence of stroke subtypes.

As a result, this study attempts to estimate the incidence rate of stroke, overall and according to stroke subtype, in crude and age-standardized form, using national hospitalization records from the years 2017 and 2018.

## Materials and Methods

### Study design and location

We conducted a study using secondary data, based on stroke records from 2017 and 2018 in Peru. The information used in this analysis comes from the hospitalization records available on the website of the National Superintendence of Health (SUSALUD) ^(9)^. This information contains data from the entire national health system: Ministry of Health, Regional Health Directorates, Social Security (ESSALUD), Armed Forces and Police Health services, and the private sector.

The databases contain information on the year and month of stroke diagnosis, the region, province and district where the event was recorded, as well as the age and sex of the hospitalized patient. In addition, the diagnosis is coded according to the International Classification of Diseases version 10 (ICD-10) and the total number of patients treated per month, which allowed us to estimate the number of hospitalized cases with the conditions of interest. However, the database does not have details of emergency care or mortality.

### Selection criteria

This analysis contains all registered cases with a diagnosis of stroke defined according to ICD-10, regardless of sex and age at which the event occurred.

### Definition of variables

Stroke was defined according to the diagnosis of any hospitalization event (new episode or recurrent episode) based on ICD-10. Thus, I60 was used to define subarachnoid hemorrhage (SAH); I61 for atraumatic intracerebral hemorrhage compatible with hemorrhagic stroke; I63 for cerebral infarction compatible with ischemic stroke; and I64 for unspecified stroke. In addition, all the cases defined above were grouped together to estimate the number of events compatible with stroke and to estimate the total incidence. The other codes were not used because they were not associated with brain damage typical of acute stroke (I65, I66 or I67) or were related to sequelae of cerebrovascular disease (I68 or I69).

Other variables of interest were sex (male, female), age classified into groups (<35, 35-44, 45-54, 45-54, 55-64, and ≥65); although the greatest interest was in the occurrence of events at age 35 or older, health sector where the case was registered (MINSA, ESSALUD, Armed Forces and Police Health, and private/other) and year of report (2017, 2018).

### Statistical analysis

STATA version 16 for Windows (StataCorp, College Station, TX, USA) and Excel 2019 (Microsoft, WA, USA) were used for analysis. Initially, the reported events of interest were tabulated by year, stroke subtype, and health sector where they were recorded. Also, the population diagnosed with stroke was briefly described.

The incidence rate of stroke was then estimated on the basis of the number of cases reported per year per 100,000 inhabitants. The denominator was defined according to the population at risk each year, based on the total population projections by calendar years, sex and age of the National Institute of Statistics and Informatics (INEI) published in 2009 ^(10)^. In this way, it was possible to estimate the total incidence, by sex and age group, as the number of stroke cases divided by the population at risk in each group of interest and specific year. The decision to use projections was based on the fact that the 2017 national census would only be useful for that year and thus, projections for 2018 would need to be used, which could drastically affect the results.

Due to the few cases of stroke in persons younger than 35 years of age, in relation to the population at risk, emphasis was placed on persons aged 35 years and older in order to compare with other international studies. The age-standardized stroke incidence rate was estimated using the direct method according to the World Health Organization (WHO) population distribution ^(11)^. Accordingly, we reported the incidence of stroke by sex and age group and their respective 95% confidence intervals (95% CI).

Finally, the same previously described steps were used to estimate the incidence rate of stroke according to subtype (SAH, hemorrhagic, ischemic, and unspecified).

### Ethical criteria

Secondary data available on the SUSALUD website were used for this analysis. The information is grouped monthly by healthcare center and without personal identifiers. Consequently, this work was not subjected to ethical review.

## Results

### Description of stroke cases

A total of 2,147,747 (994,537 in 2017 and 1,153,210 in 2018) hospitalization records were evaluated. Of which, 2,119,465 (98.7%) had the appropriate ICD-10 diagnosis (99.5% in 2017 and 97.9% in 2018) and were used in this analysis.

During 2017, a total of 10,570 stroke cases (46.6% in women) were recorded, while for 2018 there were 12,835 stroke cases (44.9% in women). Most of the stroke cases were reported by the Ministry of Health, for both 2017 and 2018 (Table 1).

Regarding stroke subtype, during 2017, 12.0% of the reported events were SAH, 16.7% were cerebral hemorrhage, 35.7% were ischemic events and 35.6% were unspecified stroke. For 2018, 10.0% were SAH, 17.7% were hemorrhagic stroke, 40.4% were ischemic stroke and 31.9% were unspecified stroke.

### Stroke incidence by sex and overall

For 2017, the crude incidence rate of stroke was 33.2 (95% CI: 32.6-33.8) per 100,000 person-years, which increased to 39.9 (95% CI: 39.2-40.6) in 2018. When estimates were standardized according to the population described by WHO, these were 38.1 (95% CI: 37.5-38.8) and 44.9 (95% CI: 44.2-45.7) for 2017 and 2018, respectively.

When only those aged 35 years and older were analyzed, the crude incidence rate of stroke increased from 80.9 (95% CI: 79.3-82.5) in 2017 to 96.7 (95% CI: 95.0-98.1) per 100,000 person-years in 2018. The standardized values were 93.9 (95% CI: 92.2-95.7) in 2017 and 109.8 (95% CI: 108.0-111.7) in 2018.

For all age groups and years studied, the incidence rate of stroke was always higher in men than in women (Table 2).

### Incidence of stroke according to subtype

For 2018, the overall incidence rate of SAH was 4.0 (95% CI: 3.8-4.2), that of hemorrhagic stroke was 7.1 (95% CI: 6.8-7.3) and that of ischemic stroke was 16.1 (95% CI: 15.7-16.6) per 100,000 person-years. On the other hand, the standardized incidence rate of SAH was 4.3 (95% CI: 4.1-4.5), that of hemorrhagic stroke was 7.8 (95% CI: 7.5-8.1) and that of ischemic stroke was 18.4 (95% CI: 17.9-18.8). While the estimates for SAH were similar to those of 2017, the incidence of hemorrhagic stroke and ischemic stroke were higher in 2018 than in 2017 (Table 3). On the other hand, the incidence of unspecified stroke increased from 11.8 (95% CI: 11.5-12.2) in 2017 to 12.7 (95% CI: 12.3-13.1) per 100,000 person-years in 2018 (Supplementary Material).

## Discussion

The results of this study described the incidence rate of stroke in Peru during 2017 and 2018, which was higher especially in those of older age and in men. Although two consecutive years may be a relatively short time to determine trends, there is also evidence of an increase in the number of stroke cases with a predominance of hemorrhagic and ischemic causes. According to stroke subtype, those of ischemic nature had a higher incidence in the two studied years, while there is still a large proportion of strokes that cannot be properly specified.

The prevalence of stroke has been estimated mostly by previous cross-sectional studies ^(12,13)^. However, according to the Global Burden of Disease Study (GBD), the crude incidence of stroke in Peru for all ages ranges between 73 and 74 per 100,000 person-years for 2017 and 2018, respectively ^(14)^. Our findings underestimate the incidence of stroke compared to the results shown by the GBD, due to the use of records identified by ICD-10 codes, whereas GBD estimates are obtained through mathematical models due to the lack of data in Peru.

Other similar studies have been conducted in similar contexts, such as countries close to Peru. For example, the study of stroke incidence in Joinville, Brazil ^(15)^, reported standardized incidence values of 90.9 per 100,000 inhabitants for 2013, but these have been decreasing since 1995, where the standardized incidence was 143.7 per 100,000 person-years. On the other hand, the standardized incidence of stroke in Durango, Mexico, has been estimated at 270.7 per 100,000 person-years by a surveillance system established in the city ^(16)^. In Ñuble, Chile, the incidence of a first stroke event has been estimated at 121.7 per 100,000 person-years; the authors collected information on hospitalization, outpatient care, and mortality records ^(17)^. In Tandil, Argentina, the standardized incidence of stroke has been estimated at 88.1 per 100,000 person-years, by means of an active search for cases in various registries ^(18)^. Finally, in Uruguay, the incidence of stroke was estimated at 181.4 cases per 100,000 person-years by means of a system for registering and capturing cases in the emergency services of the healthcare centers from the city of Rivera ^(19)^.

The findings of our study, although below all these estimates, are the first approximation to estimating the stroke incidence in the Peruvian population; even fewer studies have estimated the incidence of stroke according to subtype. Cabral *et al*. ^(20)^, using data from the Joinville study, determined that the standardized incidence of ischemic stroke (86.0 per 100,000 person-years) was much higher than the incidence of cerebral hemorrhage (12.9 per 100,000 person-years) and SAH (7.0 per 100,000 person-years). Similarly, Bahit *et al*. in Argentina ^(18)^ estimated that the standardized incidence of ischemic stroke (65.2 per 100,000 person-years) was much higher than that of hemorrhagic stroke (15.2 per 100,000 person-years) and SAH (5.2 per 100,000 person-years). Thus, the results of this study show the same trend, with ischemic subtype strokes being the most frequent and presenting the highest incidence at the population level.

These findings reinforce the need to improve the system for recording stroke cases in Peru, which could be achieved by taking the standardized WHO definition of stroke, implementing step-by-step stroke surveillance instruments (registry), and integrating information from different sources. If so, more reliable estimates could be obtained to help determine the true burden of this condition in our country. Although there is an apparent improvement in stroke registration, this must be properly sustained over time to close the epidemiological gap.

On the other hand, the higher incidence of stroke in persons aged 35 years and older implies the need for appropriate treatment of the risk factors associated with the development of these cerebrovascular events, including hypertension, type 2 diabetes mellitus and hypercholesterolemia, as well as other embolic causes, such as atrial fibrillation.

In Peru, the prevalence of hypertension in the population has been estimated to be increasing, with a reduction in the proportion of hypertensive patients who are aware of their diagnosis, as well as in those who are adequately controlled ^(21)^. Likewise, the attributable fraction of stroke due to hypertension has been estimated at almost 50% ^(22)^, which highlights the need for appropriate diagnosis and treatment of hypertensive cases. Something similar occurs with diabetes, where the prevalence has increased over the years ^(23)^ and a large proportion of people are unaware of their diagnosis and, therefore, are not adequately controlled ^(24)^. However, data on hypercholesterolemia or atrial fibrillation are almost nonexistent in our context.

The fact that a large percentage of stroke cases are classified as unspecified stroke may indicate the healthcare system’s deficiencies in making an appropriate diagnosis of these events, especially due to the absence of imaging (e.g., computed tomography), which is generally performed in tertiary care facilities ^(25)^. Thus, although a clinical diagnosis of stroke can be made, its subtype cannot be determined, which highlights the urgent need to ensure this type of diagnostic tools.

This is one of the first attempts to estimate the incidence of stroke by sex, age group and subtype in the Peruvian population. The estimates are conservative and show consistency in the two evaluated years. However, this study has some limitations that should be emphasized.

First, this study is based on the existing records of the Peruvian health system and, therefore, there may be underreporting, which could underestimate the incidence values. This problem may be due to errors in the registry, an incorrect assignment of the type of stroke when using ICD-10, or because the information reported is based on cases that entered the healthcare system (hospitalization). It is to be expected that a significant proportion of stroke cases may not have been recorded, with subsequent underestimation of the true incidence of stroke ^(25)^. This is often more apparent in rural settings ^(26)^. Thus, the increase from year to year may be a true increase in the number of stroke cases, but may also be due to a gradual improvement in recording. However, our results are conservative and within the expected values for the region, as previously reported ^(4)^.

Second, the proportion of unspecified-cause stroke cases is too large compared to other studies where such stroke type is either absent or too small. While the proportion of these unspecified cases is reduced from year to year, which may imply an improvement in health access and recording of information, this may have a large impact on the incidence of the other stroke subtypes.

Third, there is no way to properly define whether the recorded cases were a first event or a recurrent stroke. Also, there could be a small fraction of people with a history of two or more strokes in the same year.

Finally, the number of people at risk was determined using projections for our population. Its effect should be negligible, since there have been no abrupt changes in the size of the population in the studied years.

In conclusion, the results of this study show a high incidence rate of stroke in the Peruvian population, being higher in older people and in men. It is also evident that ischemic stroke cases are the most prevalent. There is a need to have an appropriate system for recording stroke cases in order to properly estimate the burden of this condition in our country.

## Supplementary Material

Available in the electronic version of the RPMESP.

Supplementary materials

## Figures and Tables

**Table 1:** Number of stroke cases according to type and origin.

Year/sub-sector	Subarachnoid Hemorrhage	Hemorrhagic Stroke	Ischemic Stroke	Not specified
2017				
MINSA	638	926	1113	1919
ESSALUD	449	567	2073	1270
Armed Forces and Police Health Services	17	63	143	58
Private or others	161	214	440	519
2018				
MINSA	631	1340	2104	2173
ESSALUD	450	627	2361	1190
Armed Forces and Police Health Services	16	52	139	40
Private or others	186	248	586	692

MINSA: Ministry of Health; ESSALUD: Social Security Health Insurance.

**Table 2:** Stroke incidence rate (per 100 000 person-years) by age and sex (2017-2018).

Year/Age group	Male		Female		Total	
	Cases	IR (95% CI)	Cases	IR (95% CI)	Cases	IR (95% CI)
2017						
Crude incidence						
<35	363	3.66 (3.28-4.03)	285	2.96 (2.62-3.30)	648	3.31 (3.06-3.57)
35-44	244	11.11 (9.71-12.51)	234	10.73 (9.36-12.11)	478	10.93 (9.95-11.90)
45-54	549	32.80 (30.05-35.54)	482	28.49 (25.94-31.03)	1031	30.63 (28.76-32.50)
55-64	990	87.18 (81.75-92.60)	714	59.93 (55.53-64.32)	1704	73.22 (69.75-76.70)
≥65	3495	347.89 (336.38-359.40)	3214	269.82 (260.50-279.13)	6709	305.54 (298.24-312.84)
Total	5641	35.39 (34.47-36.31)	4929	31.03 (30.16-31.89)	10 570	33.21 (32.58-33.84)
≥35		87.83 (85.46-90.20)		74.25 (72.12-76.39)		80.90 (79.31-82.50)
Standardized incidence						
Total		43.24 (42.24-44.27)		33.61 (32.71-34.51)		38.13 (37.45-38.81)
≥35		109.04 (106.41-111.68)		81.00 (78.77-83.23)		93.93 (92.22-95.65)
2018						
Crude incidence						
<35	360	3.62 (3.25-3.99)	321	3.33 (2.96-3.69)	681	3.48 (3.21-3.74)
55-44	296	13.29 (11.77-14.80)	255	11.53 (10.11-12.94)	551	12.41 (11.37-13.45)
45-54	687	40.12 (37.12-43.12)	539	31.16 (28.53-33.79)	1226	35.62 (33.63-37.61)
55-64	1168	99.39 (93.69-105.08)	810	65.72 (61.20-70.25)	1978	82.15 (78.53-85.77)
≥65	4555	437.29 (424.62-449.96)	3844	310.99 (301.17-320.81)	8399	368.75 (360.88-376.62)
Total	7066	43.87 (42.85-44.90)	5769	35.93 (35.00-36.85)	12 835	39.91 (39.22-40.60)
≥35		108.92 (106.31-111.52)		84.99 (82.73-87.24)		96.71 (94.99-98.43)
Standardized incidence						
Total		52.72 (51.60-53.84)		38.11 (37.12-39.11)		44.94 (44.21-45.67)
≥35		132.39 (129.51-135.26)		83.63 (81.39-85.87)		109.82 (107.99-111.65)

IR: Incidence Rate. 95%; CI: 95% Confidence Interval.

**Table 3:** Stroke incidence rate by subtypes (per 100,000 person-years) by age and type (2017 - 2018).

Year/Age group	Subarachnoid Hemorrhage		Hemorrhagic Stroke		Ischemic Stroke	
	Cases	IR (95% CI)	Cases	RI (95% CI)	Cases	RI (95% CI)
2017						
Crude incidence						
<35	217	1.11 (9.62-1.26)	213	1.09 (0.94-1.24)	77	0.39 (0.31-0.48)
35-44	128	2.93 (2.42-3.43)	99	2.26 (1.82-2.71)	112	2.56 (2.09-3.03)
45-54	236	7.01 (6.12-7.91)	209	6.21 (5.37-7.05)	285	8.47 (7.48-9.45)
55-64	251	10.79 (9.45-12.12)	306	13.15 (11.68-14.62)	620	26.64 (24.55-28.74)
≥65	433	19.72 (17.86-21.58)	943	42.95 (40.21-45.69)	2675	121.82 (117.21-126.44)
Total	1265	3.97 (3.76-4.19)	1770	5.56 (5.30-5.82)	3769	11.84 (11.46-12.22)
≥35		8.55 (8.03-9.06)		12.70 (12.07-13.33)		30.10 (29.13-31.08)
Standardized incidence						
Total		4.36 (4.13-4.59)		6.28 (6.00-6.55)		13.78 (13.37-14.19)
≥35		9.65 (9.10-10.20)		14.64 (13.96-15.31)		35.16 (34.11-36.21)
2018						
Crude incidence						
<35	236	1.20 (1.05-1.36)	246	1.26 (1.10-1.41)	89	0.45 (0.36-0.55)
35-44	125	2.82 (2.32-3.31)	123	2.77 (2.28-3.26)	170	3.83 (3.25-4.40)
45-54	227	6.59 (5.74-7.45)	265	7.70 (6.77-8.63)	408	11.85 (10.70-13.00)
55-64	241	10.01 (8.75-11.27)	390	16.20 (14.59-17.81)	706	29.32 (27.16-31.49)
≥65	454	19.93 (18.10-21.77)	1243	54.57 (51.54-57.61)	3817	167.58 (162.27-172.89)
Total	1283	3.99 (3.77-4.21)	2267	7.05 (6.76-7.34)	5190	16.14 (15.70-16.58)
≥35		8.33 (7.83-8.84)		16.08 (15.38-16.78)		40.59 (39.48-41.70)
Standardized incidence						
Total		4.31 (4.08-4.54)		7.82 (7.52-8.13)		18.36 (17.89-18.83)
≥35		9.23 (8.70-9.76)		18.14 (17.40-18.89)		46.31 (45.12-47.50)

95% CI: 95% confidence interval; IR, incidence rate

## Key Messages

### Motivation for the study

Evidence on stroke prevention and management in Peru comes mainly from international estimates or from some local hospital studies.

### Main findings

The results of this study show an approximation of the incidence rate of stroke at the population level in Peru, being higher at older age and in males. According to stroke subtype, those of ischemic nature had the highest incidence in the two studied years, while there is still a large proportion of strokes that cannot be properly specified.

### Implications

The incidence of stroke in Peru is high. A surveillance system is urgently needed to quantify the incidence of these cases and to understand their determinants.
