# Supplementary material for: Incidence Rate of Stroke in Peru
Source: Rev Peru Med Exp Salud Publica. Author manuscript; Available in PMC 2022 Jul 17. (PMC7613082; doi:10.17843/rpmesp.2021.383.7804)
Supplement: Supplementary materials [file EMS150039-supplement-Supplementary_materials.pdf]

MATERIAL SUPLEMENTARIO

S-Tabla 1. Tasa de incidencia de accidente cerebrovascular no especificado (por 100 000 personas-año) por edad (2017-2018)

Año/grupo etario	ACV no especificado	
	**Casos**	**IA (IC 95%)**
2017		
Incidencia cruda		
<35	141	0,72 (0,60-0,84)
35-44	139	3,18 (2,65--3,71)
45-54	301	8,94 (7,939,95)
55-64	527	22,65 (20,71-24,58)
≥65	2658	121,05 (116,45-125,65)
Total	3766	11,83 (11,46-12,21)
≥35		29,56 (28,60-30,52)
Incidencia estandarizada		
Total		13,71 (13,31-14,12)
≥35		34,49 (33,45-35,53)
2018		
Incidencia cruda		
<35	110	0,56 (0,46-0,67)
35-44	133	3,00 (2,49-3,50)
45-54	326	9,47 (8,44-10,50)
55-64	641	26,62 (24,56-28,68)
≥65	2885	126,66 (122,04-131,28)
Total	4095	12,73 (12,34-13,12)
≥35		31,71 (30,72-32,69)
Incidencia estandarizada		
Total		14,45 (14,03-14,86)
≥35		36,13 (35,08-37,18)

ACV: accidente cerebrovascular; IC 95%: intervalo de confianza al 95% ; TI: Tasa de incidencia.
